# Supplementary material for: Impact of Implementing Antenatal Syphilis Point-of-Care Testing on Maternal Mortality in KwaZulu-Natal, South Africa: An Interrupted Time Series Analysis
Source: Diagnostics (Basel). 2019 Dec 10;9(4):218. doi: 10.3390/diagnostics9040218 (PMC6963181; doi:10.3390/diagnostics9040218)
Supplement: Supplementary file 1 [file diagnostics-09-00218-s001.pdf]

**Table S1.** Maternal deaths by year and month for all KZN.

| | Year | Month | Number of maternal deaths in facility | Number of live births in facility | MMR per 100 000 |
|---|---|---|---|---|---|
| 1. | 2004 | 1 | 4 | 12413 | 32.22428 |
| 2. | 2004 | 2 | 26 | 11236 | 231.3991 |
| 3. | 2004 | 3 | 26 | 12807 | 203.014 |
| 4. | 2004 | 4 | 13 | 12424 | 104.6362 |
| 5. | 2004 | 5 | 13 | 13256 | 98.0688 |
| 6. | 2004 | 6 | 22 | 13542 | 162.4575 |
| 7. | 2004 | 7 | 19 | 14133 | 134.4371 |
| 8. | 2004 | 8 | 15 | 14353 | 104.5078 |
| 9. | 2004 | 9 | 25 | 16111 | 155.1735 |
| 10. | 2004 | 10 | 17 | 13048 | 130.2882 |
| 11. | 2004 | 11 | 9 | 12716 | 70.77698 |
| 12. | 2004 | 12 | 19 | 13673 | 138.96 |
| 13. | 2005 | 1 | 53 | 13785 | 384.4759 |
| 14. | 2005 | 2 | 75 | 12551 | 597.562 |
| 15. | 2005 | 3 | 96 | 14390 | 667.1299 |
| 16. | 2005 | 4 | 94 | 14083 | 667.4714 |
| 17. | 2005 | 5 | 91 | 14368 | 633.3519 |

| | | | | | |
|---|---|---|---|---|---|
| 18. | 2005 | 6 | 88 | 13830 | 636.2979 |
| 19. | 2005 | 7 | 79 | 13775 | 573.5027 |
| 20. | 2005 | 8 | 97 | 14053 | 690.2441 |
| 21. | 2005 | 9 | 113 | 15097 | 748.4931 |
| 22. | 2005 | 10 | 91 | 12702 | 716.4226 |
| 23. | 2005 | 11 | 79 | 12228 | 646.0582 |
| 24. | 2005 | 12 | 88 | 13558 | 649.0633 |
| 25. | 2006 | 1 | 28 | 14186 | 197.3777 |
| 26. | 2006 | 2 | 14 | 12824 | 109.1703 |
| 27. | 2006 | 3 | 34 | 15082 | 225.4343 |
| 28. | 2006 | 4 | 38 | 14518 | 261.744 |
| 29. | 2006 | 5 | 40 | 15040 | 265.9575 |
| 30. | 2006 | 6 | 33 | 14439 | 228.5477 |
| 31. | 2006 | 7 | 29 | 14215 | 204.0098 |
| 32. | 2006 | 8 | 36 | 14692 | 245.0313 |
| 33. | 2006 | 9 | 34 | 16110 | 211.049 |
| 34. | 2006 | 10 | 20 | 13742 | 145.5392 |
| 35. | 2006 | 11 | 25 | 12673 | 197.2698 |
| 36. | 2006 | 12 | 40 | 13709 | 291.7791 |
| 37. | 2007 | 1 | 24 | 14688 | 163.3987 |
| 38. | 2007 | 2 | 31 | 13050 | 237.5479 |

| | | | | | |
|---|---|---|---|---|---|
| 39. | 2007 | 3 | 21 | 14793 | 141.959 |
| 40. | 2007 | 4 | 31 | 14906 | 207.9699 |
| 41. | 2007 | 5 | 32 | 14962 | 213.8752 |
| 42. | 2007 | 6 | 26 | 13340 | 194.9025 |
| 43. | 2007 | 7 | 30 | 14500 | 206.8965 |
| 44. | 2007 | 8 | 26 | 13850 | 187.7256 |
| 45. | 2007 | 9 | 25 | 15787 | 158.3581 |
| 46. | 2007 | 10 | 36 | 12783 | 281.624 |
| 47. | 2007 | 11 | 20 | 12332 | 162.1797 |
| 48. | 2007 | 12 | 35 | 13589 | 257.5612 |
| 49. | 2008 | 1 | 17 | 14709 | 115.5755 |
| 50. | 2008 | 2 | 18 | 13975 | 128.8014 |
| 51. | 2008 | 3 | 20 | 15422 | 129.6849 |
| 52. | 2008 | 4 | 14 | 14822 | 94.45419 |
| 53. | 2008 | 5 | 11 | 14792 | 74.36452 |
| 54. | 2008 | 6 | 10 | 14583 | 68.573 |
| 55. | 2008 | 7 | 10 | 14970 | 66.80027 |
| 56. | 2008 | 8 | 7 | 14723 | 47.54466 |
| 57. | 2008 | 9 | 10 | 16549 | 60.42661 |
| 58. | 2008 | 10 | 1 | 13541 | 7.384979 |
| 59. | 2008 | 11 | 2 | 12624 | 15.84284 |

| | | | | | |
|---|---|---|---|---|---|
| 60. | 2008 | 12 | 0 | 14517 | 0 |
| 61. | 2009 | 1 | 21 | 14467 | 145.1579 |
| 62. | 2009 | 2 | 39 | 13342 | 292.31 |
| 63. | 2009 | 3 | 21 | 15237 | 137.8224 |
| 64. | 2009 | 4 | 29 | 13193 | 219.8135 |
| 65. | 2009 | 5 | 40 | 14093 | 283.8289 |
| 66. | 2009 | 6 | 42 | 14201 | 295.7538 |
| 67. | 2009 | 7 | 25 | 14448 | 173.0343 |
| 68. | 2009 | 8 | 23 | 14266 | 161.2225 |
| 69. | 2009 | 9 | 33 | 15528 | 212.5193 |
| 70. | 2009 | 10 | 12 | 13196 | 90.93665 |
| 71. | 2009 | 11 | 18 | 12459 | 144.4739 |
| 72. | 2009 | 12 | 21 | 13417 | 156.5179 |
| 73. | 2010 | 1 | 21 | 14307 | 146.7813 |
| 74. | 2010 | 2 | 23 | 12544 | 183.3546 |
| 75. | 2010 | 3 | 29 | 14476 | 200.3316 |
| 76. | 2010 | 4 | 18 | 14232 | 126.4755 |
| 77. | 2010 | 5 | 30 | 14254 | 210.4672 |
| 78. | 2010 | 6 | 17 | 14573 | 116.6541 |
| 79. | 2010 | 7 | 24 | 14567 | 164.756 |
| 80. | 2010 | 8 | 23 | 13650 | 168.4982 |

| | | | | | |
|---|---|---|---|---|---|
| 81. | 2010 | 9 | 26 | 15724 | 165.3523 |
| 82. | 2010 | 10 | 18 | 13035 | 138.0898 |
| 83. | 2010 | 11 | 31 | 12176 | 254.5992 |
| 84. | 2010 | 12 | 31 | 14006 | 221.3337 |
| 85. | 2011 | 1 | 43 | 13714 | 313.5482 |
| 86. | 2011 | 2 | 29 | 13165 | 220.2811 |
| 87. | 2011 | 3 | 29 | 15198 | 190.8146 |
| 88. | 2011 | 4 | 22 | 14437 | 152.3862 |
| 89. | 2011 | 5 | 30 | 15120 | 198.4127 |
| 90. | 2011 | 6 | 28 | 14327 | 195.4352 |
| 91. | 2011 | 7 | 42 | 13900 | 302.1583 |
| 92. | 2011 | 8 | 31 | 14146 | 219.1432 |
| 93. | 2011 | 9 | 35 | 15566 | 224.849 |
| 94. | 2011 | 10 | 32 | 12790 | 250.1955 |
| 95. | 2011 | 11 | 27 | 12168 | 221.8935 |
| 96. | 2011 | 12 | 30 | 13106 | 228.9028 |
| 97. | 2012 | 1 | 19 | 14418 | 131.7797 |
| 98. | 2012 | 2 | 23 | 13436 | 171.1819 |
| 99. | 2012 | 3 | 22 | 14572 | 150.9745 |
| 100. | 2012 | 4 | 20 | 14022 | 142.633 |
| 101. | 2012 | 5 | 26 | 14304 | 181.7673 |

| | | | | | |
|---|---|---|---|---|---|
| 102. | 2012 | 6 | 23 | 13797 | 166.7029 |
| 103. | 2012 | 7 | 32 | 14143 | 226.2603 |
| 104. | 2012 | 8 | 33 | 13595 | 242.7363 |
| 105. | 2012 | 9 | 30 | 14504 | 206.8395 |
| 106. | 2012 | 10 | 31 | 12287 | 252.2992 |
| 107. | 2012 | 11 | 26 | 11834 | 219.7059 |
| 108. | 2012 | 12 | 27 | 13410 | 201.3423 |
| 109. | 2013 | 1 | 16 | 14465 | 110.6118 |
| 110. | 2013 | 2 | 19 | 12657 | 150.1146 |
| 111. | 2013 | 3 | 29 | 14885 | 194.827 |
| 112. | 2013 | 4 | 21 | 13776 | 152.439 |
| 113. | 2013 | 5 | 26 | 14152 | 183.7196 |
| 114. | 2013 | 6 | 24 | 13740 | 174.6725 |
| 115. | 2013 | 7 | 13 | 13404 | 96.98598 |
| 116. | 2013 | 8 | 29 | 14222 | 203.9094 |
| 117. | 2013 | 9 | 12 | 14882 | 80.63432 |
| 118. | 2013 | 10 | 27 | 12482 | 216.3115 |
| 119. | 2013 | 11 | 25 | 12200 | 204.918 |
| 120. | 2013 | 12 | 15 | 12546 | 119.56 |
| 121. | 2014 | 1 | 21 | 13369 | 157.0798 |
| 122. | 2014 | 2 | 20 | 11066 | 180.7338 |

| | | | | | |
|---|---|---|---|---|---|
| 123. | 2014 | 3 | 21 | 14830 | 141.6049 |
| 124. | 2014 | 4 | 24 | 13765 | 174.3553 |
| 125. | 2014 | 5 | 24 | 14175 | 169.3122 |
| 126. | 2014 | 6 | 15 | 13758 | 109.0275 |
| 127. | 2014 | 7 | 22 | 13642 | 161.2667 |
| 128. | 2014 | 8 | 23 | 13626 | 168.795 |
| 129. | 2014 | 9 | 25 | 14706 | 169.9986 |
| 130. | 2014 | 10 | 15 | 12282 | 122.1299 |
| 131. | 2014 | 11 | 21 | 11494 | 182.704 |
| 132. | 2014 | 12 | 23 | 12771 | 180.0955 |
